# Association of Circulating C-C Motif Chemokine Ligand 4 to Disease Severity and Clinical Outcomes in Sepsis: A Prospective Observational Study

**DOI:** 10.3390/biomedicines14061390

**Published:** 2026-06-19

**Authors:** Hilal Sipahioglu, Koca Caliskan, Berkan Akcakaya, Sibel Kuzuguden, Hatice Kubra Zenger Ilik, Hatice Aslan Sirakaya

**Affiliations:** 1 Department of Internal Medicine, Division of Medical Intensive Care Unit, The Kayseri City Hospital, Health Science University, Kayseri 38080, Turkey; kocaclskn@hotmail.com; 2 Department of Internal Medicine, The Kayseri City Hospital, Health Science University, Kayseri 38080, Turkey; berkanakcakaya@hotmail.com (B.A.); haticekubrazenger@gmail.com (H.K.Z.I.); hasirakaya@gmail.com (H.A.S.); 3 Department of Medical Biochemistry, The Kayseri City Hospital, Health Science University, Kayseri 38080, Turkey; skuzuguden@hotmail.com

**Keywords:** sepsis, CCL4, chemokines, mortality, prognostic biomarker

## Abstract

**Background**: Sepsis is a life-threatening syndrome characterized by a dysregulated host response and organ dysfunction and remains a major cause of mortality in intensive care units (ICUs). Early risk stratification is essential for clinical management. C-C motif chemokine ligand 4 (CCL4), a pro-inflammatory chemokine involved in immune cell recruitment, may reflect the severity of systemic inflammation; however, its prognostic value in adult patients with sepsis has not been fully elucidated. **Methods**: In this prospective, single-center observational study, 75 adult patients with sepsis admitted to the ICU were enrolled. Plasma CCL4 levels were measured at admission using an enzyme-linked immunosorbent assay (ELISA). Logistic regression, receiver operating characteristic (ROC) curve analysis, DeLong testing, and reclassification analyses using net reclassification improvement (NRI) and integrated discrimination improvement (IDI) were performed. **Results**: CCL4 levels were significantly higher in non-survivors than survivors (1784 ± 752 vs. 1397 ± 528 pg/mL, *p* = 0.011). In multivariable analysis, the CCL4 (odds ratio [OR] 1.001, *p* = 0.023) and Pitt bacteremia score (OR 1.523, *p* = 0.003) were independently associated with ICU mortality. CCL4 alone showed modest discriminative performance (AUC 0.645, 95% confidence interval [CI] 0.508–0.782). However, the addition of CCL4 to clinical severity scores significantly improved discrimination, with the highest observed in the combined model (AUC 0.885). Reclassification analyses further supported the incremental prognostic value of CCL4. **Conclusions**: CCL4 is independently associated with ICU mortality in sepsis, and its integration with clinical severity scores may improve prognostic accuracy for risk stratification.

## 1. Introduction

Sepsis is a critical clinical syndrome characterized by a systemic inflammatory response and multiple organ dysfunction, leading to a substantial increase in morbidity and mortality in intensive care units. Despite advances in contemporary therapeutic strategies, it remains one of the leading causes of death among critically ill patients [1]. The clinical course of sepsis is highly heterogeneous, and some patients may experience rapid clinical deterioration accompanied by the development of multiple organ failure. Therefore, early identification of high-risk individuals and accurate prognostic assessment are of paramount importance in the clinical management of patients with sepsis.

The pathophysiology of sepsis involves a complex interplay of uncontrolled inflammatory response, immune dysregulation, microcirculatory disturbances, and endothelial dysfunction [1]. In this process, activated cytokines and chemokines regulate the migration of inflammatory cells to target tissues, thereby playing a decisive role in the development of organ damage. Chemokines are small chemotactic cytokines with a molecular weight of approximately 8–12 kDa and are classified into four main groups—CXC, CC, C, and CX3C—based on their structural characteristics. More than 40 chemokines have been identified in the human genome, and these molecules play a central role in the regulation of both innate and adaptive immune responses [2,3].

Chemokine receptors and associated surface molecules exhibit distinct expression profiles across T helper (Th) cell subsets. Th1 cells predominantly express receptors such as CCR5 and CXCR3, along with CD26, interferon-γ, and lymphocyte activation gene-3 (LAG-3), whereas Th2 cells are characterized by the expression of molecules including CCR4, CCR3, CCR8, CD62L, and CD30 [4]. These differential expression patterns underscore the critical role of the chemokine system in directing the inflammatory response. C-C motif chemokine ligand 4 (CCL4), also known as macrophage inflammatory protein-1β (MIP-1β), is a member of the CC chemokine family that exerts its effects primarily through the CCR5 receptor [5,6]. Secreted by monocytes, macrophages, dendritic cells, and T lymphocytes, CCL4 plays a key role in inflammatory cell chemotaxis and the regulation of Th1-dominant immune responses. The interaction between CCL4 and CCR5 has been suggested to enhance inflammatory cell infiltration, thereby exacerbating tissue injury [3,4].

Antimicrobial resistance and biofilm-associated bacterial persistence are increasingly recognized as important determinants of sepsis outcomes. Biofilm-forming pathogens may alter host immune recognition and modulate local chemokine responses, thereby contributing to persistent inflammation and treatment-refractory infection phenotypes [7,8]. In this context, CCL4-mediated inflammatory cell recruitment may not only reflect systemic inflammatory burden but also broader host–pathogen interaction dynamics that warrant further investigation in sepsis.

CC chemokines have been shown to be elevated in various inflammatory diseases due to their chemotactic properties that facilitate the directed migration of leukocytes to inflamed tissues [2,3]. The presence of chemokines such as macrophage inflammatory protein-1α (MIP-1α/CCL3) and macrophage inflammatory protein-1β (MIP-1β/CCL4) in atherosclerotic lesions supports their role in chronic inflammation and tissue damage [9]. Furthermore, C-C motif chemokine ligand 14 (CCL14), another member of the CC chemokine family, has been associated with renal dysfunction and mortality in patients with sepsis, with increased urinary levels reported in acute kidney injury (AKI) [10,11]. In experimental models, Kitching et al. demonstrated that crescentic glomerulonephritis is associated with a Th1-predominant immune response, highlighting the importance of the chemokine–receptor axis in inflammatory tissue injury [12].

However, studies evaluating the relationship between CCL4 levels and clinical outcomes, particularly intensive care mortality, in patients with sepsis remain limited. In addition, the potential incremental value of incorporating CCL4 into existing clinical severity scores has not been sufficiently investigated.

The aim of this study was to evaluate the association between CCL4 levels and intensive care mortality in patients with sepsis admitted to the ICU and to investigate the added prognostic value of CCL4 when integrated with established clinical severity scoring systems.

## 2. Materials and Methods

This study was designed as a single-center, prospective observational investigation conducted in the Internal Medicine Intensive Care Unit (ICU) of Kayseri City Hospital, a tertiary referral center. A total of 364 patients admitted to the ICU over a four-month period, starting from 1 November 2025, were initially screened for eligibility. Patients aged ≥18 years who fulfilled the Sepsis-3 definition of sepsis were considered eligible for inclusion, after which predefined exclusion criteria were applied.

Of these, 267 patients were excluded due to the presence of chronic kidney disease (*n =* 98), chronic liver disease *(n* = 71), malignancy (*n* = 69), rheumatologic disease (*n* = 28), or pregnancy (*n* = 1). In addition, 14 patients who declined to participate and 8 patients whose follow-up could not be completed were also excluded from the analysis. No patients under the age of 18 years were identified during the study period.

These exclusion criteria were applied to reduce biological heterogeneity and to minimize the potential confounding effects of chronic inflammatory or immunocompromised conditions on circulating chemokine responses.

**Figure 1:**
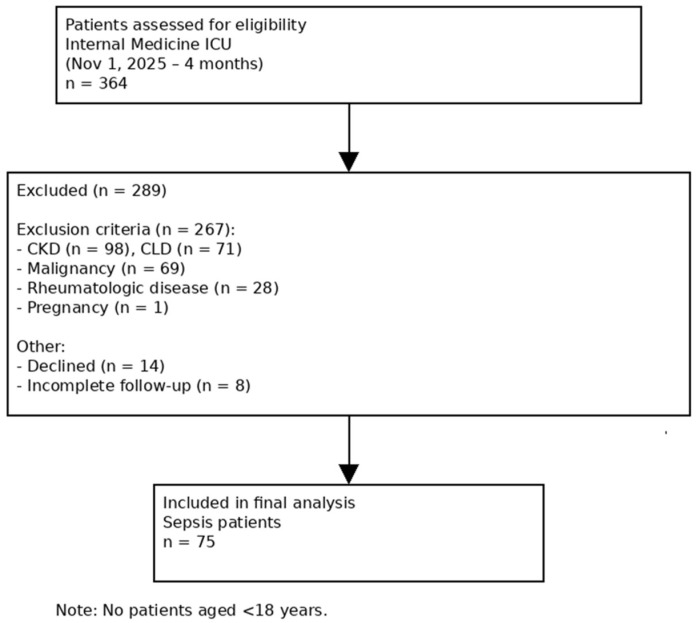
Flow diagram of patient selection and study population. Flowchart showing the screening process and exclusions leading to the final study cohort of 75 patients with sepsis. ICU, intensive care unit; CKD, chronic kidney disease; CLD, chronic liver disease.

Ultimately, a total of 75 patients with sepsis were included in the final analysis (Figure 1).

Ethical approval for the study was obtained from the Kayseri City Hospital Non-Interventional Clinical Research Ethics Committee (approval number: 2025/687). Written informed consent was obtained from the patient whenever possible; when the patient’s clinical condition precluded provision of informed consent, consent was obtained from a legally authorized representative or next of kin.

The primary outcome of the study was defined as ICU mortality. Secondary outcomes included the development of acute kidney injury (AKI) and 30-day major adverse kidney events (MAKE30).

Sepsis was diagnosed according to the Sepsis-3 criteria, defined as life-threatening organ dysfunction caused by a dysregulated host response to infection. Organ dysfunction was identified as an increase of ≥2 points in the Sequential Organ Failure Assessment (SOFA) score [1]. The diagnosis of AKI was established based on the Kidney Disease: Improving Global Outcomes (KDIGO) guidelines, using criteria of increased serum creatinine and/or decreased urine output [13].

Demographic characteristics, as well as ICU and hospital length of stay, were recorded for all patients. Laboratory evaluations included renal and hepatic function tests, inflammatory markers, hematological parameters, coagulation profiles, and selected biochemical analyses. Routine biochemical measurements, including serum creatinine, were performed in the hospital’s central laboratory using Roche diagnostic platforms (Roche Diagnostics, Mannheim, Germany) according to standard clinical laboratory procedures. Arterial blood gas analysis parameters, including lactate, partial pressure of oxygen (PaO_2_), and fraction of inspired oxygen (FiO_2_), were also recorded.

Microbiological data including infection source, culture positivity status, and isolated microorganisms were retrieved from electronic hospital records when available. Infection sources were categorized as urinary, respiratory, hepatobiliary, gastrointestinal, or soft tissue infections. Culture data included blood, urine, and endotracheal aspirate (ETA) cultures. The study was primarily designed and powered to evaluate the association between circulating CCL4 levels and ICU mortality in sepsis. Detailed subgroup analyses according to microbiological pathogen categories or antimicrobial resistance patterns were not pre-specified because the available sample size would have resulted in limited statistical power and unstable subgroup estimates.

Disease severity and organ dysfunction were assessed using the Glasgow Coma Scale (GCS) [14], Acute Physiology and Chronic Health Evaluation II (APACHE II) score [15], Pitt bacteremia score [16], SOFA score [17], and Nutrition Risk in the Critically Ill (NUTRIC) score [18]. All scoring systems were calculated within the first 24 h of ICU admission.

The NUTRIC score was developed to identify critically ill patients who are most likely to benefit from nutritional therapy and incorporates variables such as age, APACHE II and SOFA scores, comorbidities, and pre-ICU hospital length of stay [18].

MAKE30 was also evaluated and defined as a composite endpoint including all-cause mortality, initiation of renal replacement therapy (including both intermittent hemodialysis and continuous renal replacement therapy), or persistent renal dysfunction within 30 days [19]. Persistent renal dysfunction was defined as a final serum creatinine value ≥ 200% of the baseline creatinine level.

Peripheral venous blood samples were collected at ICU admission into ethylenediaminetetraacetic acid (EDTA)-containing tubes. Plasma CCL4 concentrations were measured once at the time of ICU admission. A single admission-time measurement strategy was preferred to evaluate the potential utility of CCL4 as an early prognostic biomarker during the initial phase of ICU assessment in sepsis. Samples were centrifuged at 3000 rpm for 5 min to obtain plasma fractions, which were stored at −80 °C until analysis. CCL4 concentrations were determined using a commercially available sandwich enzyme-linked immunosorbent assay (ELISA) kit (Reed Biotech, Wuhan, China, RE1010H) according to the manufacturer’s instructions. The assay had a reported analytical range of 31.25–2000 pg/mL and a minimum detectable concentration of 18.75 pg/mL.

All measurements were performed in triplicate, and concentrations were calculated using standard calibration curves. Analytical performance was confirmed by excellent linearity of the standard curve (r = 0.998), while both intra-assay and inter-assay coefficients of variation were below 5%. Results were reported in pg/mL.

The sample size was determined based on an a priori power analysis assuming a moderate effect size (Cohen’s d ≈ 0.65), 80% statistical power, and a two-sided alpha level of 0.05, resulting in a minimum required sample size of 34 patients per group. Given the exploratory biomarker-focused nature of the study and the limited availability of prior adult sepsis data specifically evaluating circulating CCL4 levels, a moderate standardized effect size assumption was considered methodologically acceptable during the study planning phase.

Post hoc power was estimated using G*Power software (version 3.1.9.7; Heinrich Heine University Düsseldorf, Düsseldorf, Germany) based on the observed difference in CCL4 levels between survivors and non-survivors. The observed standardized effect size was Cohen’s d = 0.62, which was close to the initially assumed effect size of 0.65, yielding an achieved power of 73.2% at a two-sided alpha level of 0.05.

Statistical analyses were performed using SPSS software (version 22.0; IBM Corp., Armonk, NY, USA). The distribution of continuous variables was assessed using the Kolmogorov–Smirnov test. Normally distributed variables were expressed as mean ± standard deviation (SD), while non-normally distributed variables were presented as median (IQR, 25th–75th percentile). For comparisons between two groups, the independent samples *t*-test was used for normally distributed variables, and the Mann–Whitney U test was applied for non-normally distributed variables. Categorical variables were expressed as counts and percentages and compared using the chi-square test or Fisher’s exact test, as appropriate.

Correlations between continuous variables were evaluated using Pearson or Spearman correlation analysis. The prognostic performance of CCL4 levels was assessed using receiver operating characteristic (ROC) curve analysis, and the area under the curve (AUC) was calculated with 95% confidence intervals (CIs). The performance of combined models incorporating clinical scores and CCL4 was compared using ROC analysis, and differences between AUCs were evaluated using the DeLong test.

The incremental value of adding CCL4 to clinical scoring systems was further assessed using net reclassification improvement (NRI) and integrated discrimination improvement (IDI) analyses. Risk factors were initially evaluated using univariable logistic regression analysis, and variables found to be significant were included in the multivariable model.

Patients with incomplete key clinical or laboratory variables required for the primary analyses were excluded prior to statistical evaluation. Accordingly, no additional imputation procedures were applied in the present study.

Results were reported as odds ratios (ORs) with 95% CIs, and a *p*-value < 0.05 was considered statistically significant.

## 3. Results

A total of 75 patients with sepsis admitted to the internal medicine intensive care unit (ICU) were included in the study. The demographic characteristics and clinical severity scores of the patients are presented in Table 1. No significant differences were observed between survivors and non-survivors in terms of age and sex distribution (*p* > 0.05). Similarly, the presence of acute kidney injury (AKI) did not differ significantly between the two groups (*p* = 0.255). Patients who died in the ICU (38.7%) required advanced respiratory support and vasopressor therapy significantly more frequently than survivors (*p* = 0.013 and *p* < 0.001, respectively). In the assessment of clinical severity scores, the Glasgow Coma Scale (GCS) was significantly lower in the non-survivor group (*p* = 0.002), whereas Acute Physiology and Chronic Health Evaluation II (APACHE II), Sequential Organ Failure Assessment (SOFA), Pitt bacteremia, and Nutrition Risk in the Critically Ill (NUTRIC) scores were all significantly higher (*p* ≤ 0.005 for all). In addition, the incidence of 30-day major adverse kidney events (MAKE30) was markedly higher in the non-survivor group (*p* < 0.001). There were no significant differences between the groups in terms of ICU and hospital length of stay (*p* > 0.05).

Microbiological characteristics of the cohort are summarized in Supplementary Table S5. Urinary tract and respiratory tract infections were the most common sources of infection. Culture-negative sepsis constituted a substantial proportion of the cohort. Among culture-positive patients, the most frequently isolated microorganisms were *Escherichia coli* and *Klebsiella pneumoniae*. Occasional fungal pathogens, including *Candida* species, as well as non-fermentative Gram-negative organisms, were also identified.

Laboratory findings are presented in Table 2. Blood urea nitrogen (BUN), aspartate aminotransferase (AST), and C-reactive protein (CRP) levels were significantly higher in the non-survivor group (*p* = 0.022, *p* = 0.035, and *p* = 0.029, respectively). By contrast, albumin levels were significantly lower among non-survivors (*p* = 0.010). Procalcitonin levels were also significantly elevated in patients who developed ICU mortality (*p* = 0.045). However, no significant differences were observed between the groups in terms of glomerular filtration rate (GFR), alanine aminotransferase (ALT), or other routine biochemical parameters (*p* > 0.05). Notably, levels of the primary biomarker of interest, C-C motif chemokine ligand 4 (CCL4), were significantly higher in the non-survivor group (*p* = 0.011). Although lactate levels tended to be higher among non-survivors, this difference did not reach statistical significance (*p* = 0.061).

Correlation analyses between CCL4 levels and selected clinical and laboratory parameters are presented in Supplementary Table S3. No statistically significant correlations were observed between CCL4 levels and conventional inflammatory markers, renal function parameters, vasopressor requirement, or major clinical severity scores.

Univariable and multivariable logistic regression analyses were conducted to determine independent predictors of ICU mortality (Table 3).

In univariable analysis, Acute Physiology and Chronic Health Evaluation II (APACHE II) score (*p* = 0.002), Pitt bacteremia score (*p* < 0.001), Sequential Organ Failure Assessment (SOFA) score (*p* = 0.001), and C-C motif chemokine ligand 4 (CCL4) levels (*p* = 0.016) were significantly associated with ICU mortality.

In the multivariable model, the Pitt bacteremia score remained an independent predictor of ICU mortality (*p* = 0.003). CCL4 levels were also independently associated with ICU mortality (*p* = 0.023). The SOFA score demonstrated borderline significance in the multivariable analysis (*p* = 0.052), whereas the APACHE II score was not identified as an independent predictor (*p* = 0.405).

The overall logistic regression model was statistically significant (Omnibus χ^2^ = 38.741, *p* < 0.001). The Hosmer–Lemeshow goodness-of-fit test demonstrated acceptable calibration of the multivariable logistic regression model (χ^2^ = 3.882, *p* = 0.868).

The predictive performance of CCL4 for various clinical outcomes was further evaluated using receiver operating characteristic (ROC) curve analysis (Table 4).

The predictive performance of CCL4 for acute kidney injury (AKI) and related renal outcomes was limited. The area under the curve (AUC) values for AKI prediction ranged between 0.458 and 0.563, while the AUC for 30-day major adverse kidney events (MAKE30) was 0.550.

In contrast, CCL4 demonstrated a stronger predictive performance for mortality outcomes. The AUC for ICU mortality was 0.645 (*p* = 0.038), whereas the AUC for overall mortality was 0.662 (*p* = 0.006).

The predictive performances of clinical severity scores, PCT, CCL4, and their combined models for ICU mortality are presented in Table 5.

Among the clinical severity scores evaluated individually, the highest predictive performance was observed with the Pitt bacteremia score (AUC = 0.827), followed by the Sequential Organ Failure Assessment (SOFA) score (AUC = 0.756) and the Acute Physiology and Chronic Health Evaluation II (APACHE II) score (AUC = 0.704). The AUC value of C-C motif chemokine ligand 4 (CCL4) alone was 0.645.

The addition of CCL4 to clinical severity scores resulted in improved model performance. The combined model incorporating the Pitt bacteremia score and CCL4 yielded an AUC of 0.861, while the combination of SOFA score and CCL4 increased the AUC to 0.801. A model including APACHE II, SOFA, and Pitt bacteremia scores demonstrated an AUC of 0.854, whereas the full model integrating these scores with CCL4 achieved the highest discriminative performance (AUC = 0.885).

**Figure 2:**
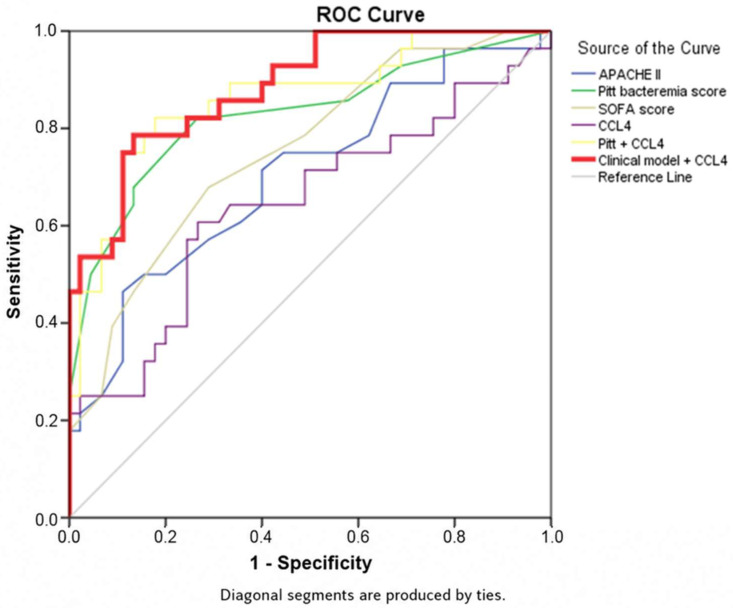
Receiver operating characteristic (ROC) curves of clinical severity scores and CCL4 for predicting ICU mortality. ROC curves demonstrating the discriminative performance of APACHE II, SOFA score, Pitt bacteremia score, and CCL4 for predicting ICU mortality. Among individual variables, the Pitt bacteremia score showed the highest predictive performance. The addition of CCL4 to clinical severity scores improved model discrimination, with the combined models (Pitt + CCL4 and clinical model + CCL4) demonstrating superior performance compared to individual predictors. The diagonal reference line represents no discriminative ability. Abbreviations: ROC, receiver operating characteristic; AUC, area under the curve; ICU, intensive care unit; APACHE II, Acute Physiology and Chronic Health Evaluation II; SOFA, Sequential Organ Failure Assessment; CCL4, C-C motif chemokine ligand 4.

Receiver operating characteristic (ROC) curves for these models are presented in Figure 2.

Table 6 summarizes the incremental changes in model discrimination following the addition of PCT and CCL4. The addition of PCT increased the AUC of the APACHE II, SOFA, Pitt bacteremia score, and combined clinical models by 0.009, 0.027, 0.012, and 0.002, respectively. The corresponding increases associated with CCL4 were 0.033, 0.045, 0.034, and 0.031, respectively.

To explore the relationship between the two biomarkers further, a multivariable logistic regression model incorporating both CCL4 and procalcitonin (PCT) was constructed. In this model, CCL4 remained independently associated with ICU mortality (OR 1.001, 95% CI 1.000–1.002, *p* = 0.017), whereas PCT was not independently associated with mortality (OR 1.001, 95% CI 0.980–1.023, *p* = 0.924). Although both biomarkers demonstrated comparable standalone discriminative performance, only CCL4 retained independent prognostic significance when evaluated simultaneously with PCT.

To assess the incremental predictive value of C-C motif chemokine ligand 4 (CCL4) when added to clinical models, differences between receiver operating characteristic (ROC) curves were evaluated using the DeLong test (Table 7).

The addition of C-C motif chemokine ligand 4 (CCL4) to the Sequential Organ Failure Assessment (SOFA) score resulted in a significant increase in predictive performance, with the AUC improving from 0.756 to 0.801 (ΔAUC = 0.046, *p* = 0.012). Similarly, incorporating CCL4 into the Acute Physiology and Chronic Health Evaluation II (APACHE II) score significantly enhanced model performance (ΔAUC = 0.033, *p* = 0.004).

In the combined clinical model, the inclusion of CCL4 also led to a significant improvement in predictive performance (ΔAUC = 0.031, *p* = 0.041). Although the addition of CCL4 to the Pitt bacteremia score increased the AUC, this improvement did not reach statistical significance (*p* = 0.058).

To further evaluate the incremental contribution of CCL4 to mortality prediction, Net Reclassification Improvement (NRI) and Integrated Discrimination Improvement (IDI) analyses were performed (Table 8).

The addition of C-C motif chemokine ligand 4 (CCL4) to the Pitt bacteremia score significantly improved risk reclassification, as demonstrated by both Net Reclassification Improvement (NRI) and Integrated Discrimination Improvement (IDI) analyses (*p* = 0.038 and *p* = 0.027, respectively).

Similarly, incorporating CCL4 into the Sequential Organ Failure Assessment (SOFA) score resulted in a significant improvement in risk classification (*p* = 0.021 and *p* = 0.018, respectively).

The addition of CCL4 to the Acute Physiology and Chronic Health Evaluation II (APACHE II) score also enhanced mortality prediction performance (*p* = 0.029 and *p* = 0.022, respectively).

Furthermore, integrating CCL4 into the combined clinical model led to a significant improvement in risk reclassification (*p* = 0.041 and *p* = 0.031, respectively).

## 4. Discussion

In this study, the potential role of C-C motif chemokine ligand 4 (CCL4) in predicting mortality and clinical outcomes in critically ill patients was evaluated. The main findings can be summarized in three key points. First, CCL4 levels were significantly higher in patients with fatal outcomes compared to survivors. Second, in multivariable logistic regression analysis, CCL4 levels, together with the Pitt bacteremia score, were identified as independent predictors of ICU mortality. Third, and most importantly, the addition of CCL4 to existing clinical severity scores significantly improved mortality prediction performance. Although the predictive performance of CCL4 alone was moderate, its integration with clinical scores markedly enhanced the discriminative ability of the models. Notably, the full model combining APACHE II, Sequential Organ Failure Assessment (SOFA), and Pitt bacteremia scores with CCL4 demonstrated the highest predictive performance. Furthermore, DeLong test comparisons, along with Net Reclassification Improvement (NRI) and Integrated Discrimination Improvement (IDI) analyses, confirmed that the inclusion of CCL4 significantly improved mortality risk stratification. These findings suggest that CCL4 may serve as a complementary biomarker reflecting the inflammatory response and enhancing prognostic accuracy in critically ill patients.

In our study, the ICU mortality rate was approximately 39%, which is consistent with previously reported mortality rates in sepsis. Prior studies have reported ICU sepsis mortality rates ranging between 30% and 60% [20,21,22,23]. This wide variation may be attributed to differences in patient characteristics, disease severity, comorbidities, and treatment strategies. The consistency of our findings with the existing literature supports the clinical representativeness of our cohort.

In the present cohort, urinary and respiratory tracts were the predominant sources of infection, while culture-negative sepsis also accounted for a considerable proportion of patients. Among culture-positive cases, Gram-negative pathogens such as *Escherichia coli* and *Klebsiella pneumoniae* were the most frequently isolated microorganisms. Although microbiological characterization data were available, the study was not specifically powered for pathogen-stratified or antimicrobial resistance-based subgroup analyses. Given the relatively limited sample size and the low frequency of several individual pathogens, such analyses could have produced inconsistent estimates and reduced statistical reliability. Furthermore, detailed antimicrobial susceptibility patterns and appropriateness of empirical antimicrobial therapy were not systematically available for all patients. Larger multicenter studies are therefore needed to clarify the relationship between CCL4 levels, microbiological phenotypes, antimicrobial resistance patterns, and clinical outcomes in sepsis.

No significant differences were observed between survivors and non-survivors in terms of age and sex. Although the association between age and sepsis mortality has been demonstrated in previous studies [20,24], similar to our findings, some reports have not identified a significant relationship [23,25]. This may be explained by the predominance of older patients in the study population and the relatively homogeneous age distribution between groups. The impact of sex on sepsis prognosis also remains inconclusive, with some studies reporting no association and others suggesting higher mortality in males [22,23,24,25].

Laboratory findings indicated that non-survivors had worse renal function and higher levels of inflammatory markers, consistent with previous studies demonstrating an association between renal dysfunction and mortality in sepsis [20]. Hypoalbuminemia, as an indicator of inflammation and nutritional status, has also been associated with poor prognosis in critically ill patients [22].

Existing literature suggests that CCL4 levels in healthy individuals typically range between 150 and 400 pg/mL, whereas levels may increase by approximately 5–10-fold in sepsis and septic shock [26,27,28]. However, studies evaluating CCL4 levels in sepsis remain limited. In the study by Jekarl et al., the high standard deviation (±1870 pg/mL) suggests a markedly heterogeneous and right-skewed distribution of CCL4 levels [27]. Similarly, Mosevoll et al. reported a wide distribution of CCL4 levels in sepsis and bacteremia, with values reaching up to 0.47 ng/mL (0.24–6.9), corresponding to approximately 6900 pg/mL [29]. These findings indicate a pronounced increase in inflammatory chemokine response during sepsis. In our study, CCL4 levels were markedly elevated in sepsis patients and were higher in those with fatal outcomes. Although our findings suggest higher absolute levels compared to some reports, the distribution appeared narrower. These differences may be attributed to variations in patient populations, disease severity, ICU-specific characteristics, and analytical methodologies used for cytokine measurement. In particular, the inclusion of critically ill ICU patients with relatively similar disease severity may explain both the higher levels and the narrower distribution observed in our cohort.

Studies across different disease groups indicate that CCL4 is not merely an inflammatory marker but also an active mediator involved in the regulation of the immune microenvironment [30,31,32]. In these studies, elevated CCL4 levels have been associated with adverse clinical outcomes, suggesting that this chemokine may reflect dysregulated and maladaptive immune activation. Our findings, demonstrating an association between CCL4 levels and mortality, support this concept.

Beyond systemic inflammation alone, emerging evidence suggests that chemokine signaling may also interact with pathogen persistence mechanisms in sepsis. Biofilm-forming microorganisms can alter immune recognition and modulate local inflammatory responses, potentially contributing to persistent or treatment-refractory infection states [33,34]. Although CCL4 does not directly mediate antimicrobial activity, its role in inflammatory cell recruitment may indirectly reflect complex host–pathogen interactions observed in severe sepsis. Nevertheless, these mechanisms remain incompletely understood and require further mechanistic investigation.

CCL4 has also been implicated in endothelial injury and vascular inflammation [35,36,37]. Considering that endothelial dysfunction is a key mechanism underlying organ failure in sepsis, the role of CCL4 in this process is of particular relevance. Our results further support the hypothesis that elevated CCL4 levels may reflect pathophysiological processes related to endothelial damage and systemic inflammation.

In sepsis-associated acute respiratory distress syndrome (ARDS), elevated CCL4 levels have been shown to reflect a pronounced proinflammatory phenotype associated with lung infiltration [38]. Experimental studies have demonstrated that apoptotic bodies released from M1 macrophages can activate neighboring macrophages via CCL4 signaling, amplifying the inflammatory response and shifting the M1/M2 balance toward a proinflammatory state [39]. Inhibition of this pathway has been proposed as a potential therapeutic strategy. Similarly, CCL4 has been identified as a key proinflammatory chemokine in kidney diseases, with evidence suggesting that targeting CCL4 pathways may attenuate acute kidney injury and prevent progression to chronic kidney disease [40]. Increased CCL4 levels have also been reported in rheumatoid arthritis, where it may serve as a marker of immune dysregulation and a potential therapeutic target [41].

Procalcitonin (PCT) is one of the most extensively investigated biomarkers in sepsis and is widely used in clinical practice for supporting the diagnosis of bacterial infection, assessing disease severity, and guiding antimicrobial stewardship strategies. Previous studies and meta-analyses have demonstrated that elevated PCT concentrations are associated with adverse outcomes and increased mortality risk in septic patients, although its prognostic performance is generally considered moderate when used as a standalone marker [42,43]. In the present study, PCT levels were significantly higher among non-survivors and demonstrated modest discriminative performance for ICU mortality. However, when CCL4 and PCT were simultaneously entered into a multivariable model, only CCL4 remained independently associated with ICU mortality, whereas PCT did not retain statistical significance. Notably, correlation analyses revealed no significant association between CCL4 and PCT levels (Supplementary Table S3), suggesting that these biomarkers may reflect distinct biological dimensions of the septic response. While PCT primarily reflects pathogen-driven inflammatory activation and bacterial burden, CCL4 is more closely linked to leukocyte recruitment, chemokine-mediated immune signaling, and host inflammatory regulation. The absence of a significant correlation between the two biomarkers, together with the independent prognostic contribution of CCL4, supports the hypothesis that CCL4 may provide complementary information beyond that captured by conventional inflammatory markers. Importantly, these findings should not be interpreted as evidence of superiority of CCL4 over PCT, as the present study was not specifically designed for direct biomarker comparison and serial measurements were not available. Rather, they suggest that CCL4 may capture prognostically relevant immunopathological processes that are not fully represented by established biomarkers currently used in routine sepsis management.

Collectively, these findings suggest that CCL4 reflects the magnitude of systemic inflammatory response rather than organ-specific injury. In our study, early measurement of CCL4 enabled assessment of the peak inflammatory phase, contributing to its discriminative performance in mortality prediction.

Studies on coronavirus infections have similarly demonstrated that CCL4 plays an active role in regulating immune cell migration and amplifying inflammatory responses [44], further supporting its role as a mediator of maladaptive hyperinflammation.

As expected, non-survivors had lower Glasgow Coma Scale (GCS) scores and higher APACHE II, SOFA, and Pitt bacteremia scores. These findings are consistent with the established role of these scoring systems in predicting mortality in sepsis and critical illness [1,20]. Recent studies have shown that SOFA and APACHE II scores generally demonstrate moderate predictive performance, with AUC values typically below 0.85. For instance, Li et al. reported similar performance for SOFA (AUC 0.809) and APACHE II (AUC 0.806) in predicting 28-day mortality [45]. Likewise, other studies have reported AUC values of approximately 0.80 for APACHE II [46]. Although the Pitt bacteremia score may demonstrate relatively higher performance in certain bacteremia-focused cohorts, its discriminative ability is not consistently superior [47]. Furthermore, in unselected sepsis populations, predictive performance may decline further, with reported AUC values of 0.738 for SOFA and 0.685 for Pitt bacteremia score [48]. Taken together, these findings suggest that traditional clinical scores alone may be insufficient for achieving high discriminative performance and that integration with novel biomarkers may improve prognostic accuracy. In our study, the Pitt bacteremia score demonstrated the highest discriminative performance among individual clinical scores, consistent with its ability to capture infection-related clinical deterioration.

The association between CCL4 levels and mortality highlights its prognostic relevance. However, the moderate discriminative performance of CCL4 alone suggests that a single biomarker may be insufficient in a heterogeneous syndrome such as sepsis. Importantly, the addition of CCL4 to clinical severity scores significantly improved prognostic performance, particularly in combined models where discriminative ability was markedly enhanced. DeLong test comparisons, along with NRI and IDI analyses, further demonstrated that incorporating CCL4 into existing models significantly improved risk stratification. These findings support the role of CCL4 as a complementary biomarker when used alongside established clinical scores.

Interestingly, CCL4 levels did not demonstrate significant correlations with conventional inflammatory markers or major clinical severity scores in the present cohort. This finding may suggest that CCL4 reflects distinct immunopathological processes that are not fully represented by routinely used inflammatory biomarkers or composite clinical scoring systems. The absence of strong linear correlations, despite the observed association with mortality and incremental prognostic improvement in combined models, further supports the possibility that CCL4 may provide complementary biological information in the complex pathophysiology of sepsis.

Evidence from Mendelian randomization studies further supports a potential causal role of inflammatory mediators in sepsis pathogenesis. Two-sample Mendelian randomization analyses have demonstrated that genetically determined CCL4 levels are significantly associated with both sepsis susceptibility and sepsis-related mortality [49,50]. These findings, supported by inverse variance-weighted analyses and sensitivity testing, reduce the likelihood that observed associations are due to confounding. Moreover, the relatively consistent association of CCL4 compared to other cytokines suggests a potentially distinct role in sepsis pathophysiology. Taken together, these data support the biological plausibility of our findings and suggest that the observed association between CCL4 and ICU mortality may reflect an underlying causal mechanism.

The transition from hyperinflammation to immunoparalysis in sepsis represents a complex pathophysiological process underlying organ failure and therapeutic challenges [21]. Previous studies have suggested that CCL4 may reflect the intensity of cytokine-mediated inflammatory response [51]. In a study by Nowak et al. in pediatric septic shock patients, admission CCL4 levels were reported as a potential predictor of mortality, with an AUC of 0.698 [52]. The comparable discriminative performance observed in our study further supports the potential utility of CCL4 as a prognostic biomarker across different patient populations.

Although the present study was conducted in a single tertiary referral ICU, several methodological features strengthen the internal consistency of the findings. The prospective design, standardized biomarker measurements, and relatively homogeneous critically ill sepsis population allowed a more controlled evaluation of the association between circulating CCL4 levels and clinical outcomes. Furthermore, all patients were managed within the same institutional treatment framework, potentially reducing variability related to local clinical practices and laboratory procedures. Nevertheless, regional differences in pathogen distribution, antimicrobial resistance patterns, and sepsis management strategies may influence the external generalizability of the results. Therefore, larger multicenter studies involving diverse ICU populations are required to validate these findings across different healthcare settings.

This study has other limitations. First, it was conducted in a single center with a relatively limited sample size, which may restrict the generalizability of the findings and necessitates validation in larger, multicenter cohorts. Second, CCL4 levels were measured at a single time point upon ICU admission, precluding assessment of temporal dynamics. Third, given the heterogeneity of sepsis, potential confounding factors such as infection source, causative pathogens, and treatment strategies could not be fully controlled. In addition, patients with chronic kidney disease, chronic liver disease, malignancy, and rheumatologic disorders were excluded to reduce biological heterogeneity and minimize potential confounding effects on chemokine regulation. Consequently, the applicability of our findings to these patient populations remains uncertain. Fourth, the predictive models developed in this study were not externally validated in an independent cohort. Finally, only a limited number of inflammatory biomarkers were evaluated; future studies incorporating broader cytokine and chemokine panels may provide a more comprehensive understanding of the role of CCL4 in sepsis pathophysiology. Furthermore, immunocompromised and chronically inflamed patient populations may exhibit altered inflammatory and chemokine responses; therefore, future studies specifically evaluating these subgroups may provide additional insight into the clinical utility of CCL4 in sepsis. From a clinical perspective, the integration of CCL4 into existing severity scoring systems may facilitate early risk stratification and support decision-making in critically ill patients.

## 5. Conclusions

In conclusion, this study demonstrates that C-C motif chemokine ligand 4 (CCL4) levels are associated with mortality in critically ill patients and that the addition of CCL4 to existing clinical severity scores significantly improves mortality prediction performance. Although CCL4 alone exhibits moderate discriminative ability, its integration with clinical scores enhances prognostic accuracy, highlighting its value as a complementary biomarker. Therefore, the measurement of CCL4 may be considered as an adjunct to established clinical scoring systems to improve risk stratification and strengthen prognostic assessment in critically ill patients.

## Figures and Tables

**Table 1:** Demographic characteristics and clinical severity scores according to ICU mortality.

Variable	Total (*n* = 75)	Survivors (*n* = 46)	Non-Survivors (*n* = 29)	*p*-Value
Age (years)	74.7 ± 13.4	74.4 ± 13.2	75.0 ± 13.8	0.860
Sex (Male/Female), *n* (%)	37 (49.3)/38 (50.7)	22 (47.8)/24 (52.2)	15 (51.7)/14 (48.3)	0.750
Acute kidney injury, *n* (%)	43 (57.3)	24 (52.2)	19 (65.5)	0.255
Advanced respiratory support *, *n* (%)	14 (18.7)	4 (8.7)	10 (34.5)	0.013
Vasopressor requirement, *n* (%)	19 (25.3)	5 (10.9)	14 (48.3)	<0.001
Glasgow Coma Scale	11.4 ± 4.0	12.7 ± 2.8	9.4 ± 4.8	0.002
APACHE II score	22.5 (17.5–29.25)	20 (16–26)	29 (21–32)	0.002
SOFA score	4 (2–5)	3 (2–5)	5 (4–8)	<0.001
Pitt bacteremia score	3.90 ± 3.67	2.18 ± 2.24	6.68 ± 3.86	<0.001
NUTRIC score	5.49 ± 1.56	5.09 ± 1.43	6.14 ± 1.56	0.004
MAKE30, *n* (%)	35 (47.9%)	10 (22.2%)	25 (89.3%)	<0.001
ICU stay (days)	6 (3–11)	6 (3–9)	6 (3–13)	0.720
Hospital stay (days)	13 (6–22)	16 (7–30)	7 (3–18)	0.088

Data are presented as mean ± standard deviation or median (IQR, 25th–75th percentile) according to data distribution. Categorical variables are expressed as number (percentage). * Advanced respiratory support included endotracheal mechanical ventilation, high-flow oxygen therapy, and CPAP/BiPAP support. Percentages were calculated using available data for each variable. Missing values were excluded from percentage calculations. Abbreviations: GCS, Glasgow Coma Scale; APACHE II, Acute Physiology and Chronic Health Evaluation II; SOFA, Sequential Organ Failure Assessment; NUTRIC, Nutrition Risk in Critically Ill; MAKE30, Major Adverse Kidney Events at 30 days.

**Table 2:** Key laboratory parameters and biomarker levels according to ICU mortality.

Variable (Unit)	Total	Survivors	Non-Survivors	*p*-Value
GFR (mL/min/1.73 m^2^)	36 (16–72)	41 (19–80)	24 (16–52)	0.066
BUN (mg/dL)	37 (22–57)	30 (17–56)	49 (29–66)	0.022
Creatinine (mg/dL)	1.66 (0.96–2.81)	1.51 (0.76–2.48)	2.37 (1.12–3.01)	0.053
AST (U/L)	29 (19–55)	26 (17–54)	38 (24–55)	0.035
ALT (U/L)	19 (10–44)	19 (9–41)	22 (14–51)	0.441
Albumin (g/dL)	3.0 ± 0.6	3.1 ± 0.5	2.8 ± 0.6	0.010
CRP (mg/L)	122.1 (49.1–228)	97.8 (47.4–168.7)	166.5 (111.1–238)	0.029
LDH (U/L)	326 (254–405)	326 (254–366)	344 (259–470)	0.186
Neutrophil count (cells/µL)	9670 (5510–15,520)	9470 (5080–15,000)	10,500 (6270–15,520)	0.303
Lymphocyte count (cells/µL)	890 (530–1470)	990 (670–1380)	720 (530–1470)	0.539
Platelets (×10^3^/µL)	192 (100–261)	204 (113–258)	165 (99–275)	0.846
INR (-)	1.25 (1.15–1.46)	1.21 (1.13–1.45)	1.34 (1.20–1.55)	0.150
Procalcitonin (ng/mL)	0.82 (0.22–4.33)	0.54 (0.17–2.27)	1.26 (0.61–4.59)	0.045
NT-proBNP (pg/mL)	3368 (1299–6703)	3257 (1374–6804)	4581 (1262–5414)	0.804
Lactate (mmol/L)	1.8 (1.1–2.6)	1.5 (0.9–2.5)	2.0 (1.4–2.9)	0.061
CCL4 (pg/mL)	1551 ± 645	1397 ± 528	1784 ± 752	0.011

Data are presented as mean ± standard deviation or median (IQR, 25th–75th percentile) according to data distribution. Abbreviations: GFR: Glomerular filtration rate; BUN: Blood urea nitrogen; AST: Aspartate aminotransferase; ALT: Alanine aminotransferase; CRP: C-reactive protein; LDH: Lactate dehydrogenase; INR: International normalized ratio; NT-proBNP: N-terminal pro-B-type natriuretic peptide; CCL4: C-C motif ligand 4.

**Table 3:** Univariable and multivariable logistic regression analysis of factors associated with ICU mortality.

Variable	Univariable Analysis			Multivariable Analysis		
	OR	*p*-Value	95% CI	OR	*p*-Value	95% CI
APACHE II score	1.109	0.002	1.039–1.185	0.958	0.405	0.865–1.060
Pitt bacteremia score	1.565	<0.001	1.272–1.925	1.523	0.003	1.158–2.003
SOFA score	1.535	0.001	1.194–1.973	1.394	0.052	0.997–1.950
CCL4 (pg/mL)	1.001	0.016	1.000–1.002	1.001	0.023	1.000–1.003

Results are presented as odds ratios (ORs) with 95% confidence intervals (CIs). Given the unit scale of CCL4 (pg/mL), odds ratios were also interpreted per 100 pg/mL increase to enhance clinical interpretability; this corresponds to an approximately 10% higher odds of ICU mortality. Abbreviations: OR, odds ratio; CI, confidence interval; APACHE II, Acute Physiology and Chronic Health Evaluation II; SOFA, Sequential Organ Failure Assessment; CCL4, C-C motif chemokine ligand 4.

**Table 4:** ROC Analysis of Day-1 CCL4 for Predicting Clinical Outcomes.

Outcome	AUC (95% CI)	*p*-Value
AKI (Day 1, KDIGO stages 1–3)	0.538 (0.406–0.670)	0.578
AKI (Day 3, KDIGO stages 1–3)	0.563 (0.428–0.698)	0.361
Moderate–severe AKI (Day 0, KDIGO stages 2–3)	0.458 (0.314–0.601)	0.544
Moderate–severe AKI (Day 3, KDIGO stages 2–3)	0.461 (0.283–0.640)	0.632
Δ Creatinine ≥ 0.3 mg/dL	0.610 (0.449–0.770)	0.180
MAKE30	0.550 (0.413–0.686)	0.467
ICU mortality	0.645 (0.508–0.782)	0.038
Overall mortality	0.662 (0.529–0.795)	0.006

Receiver operating characteristic (ROC) curves were used to evaluate the predictive performance of CCL4 measured on day 1 for clinical outcomes. Abbreviations: AKI, acute kidney injury; KDIGO, Kidney Disease: Improving Global Outcomes; MAKE30, major adverse kidney events within 30 days; AUC, area under the curve; CI, confidence interval.

**Table 5:** Receiver Operating Characteristic (ROC) Analysis of Clinical Scores and CCL4 for Predicting ICU Mortality.

Model	AUC	SE	95% CI	*p*-Value
APACHE II score	0.704	0.064	0.578–0.830	0.004
Pitt bacteremia score	0.827	0.054	0.722–0.933	<0.001
SOFA score	0.756	0.058	0.643–0.869	<0.001
CCL4	0.645	0.070	0.508–0.782	0.038
APACHE II + CCL4	0.737	0.061	0.617–0.856	0.001
Pitt bacteremia score + CCL4	0.861	0.046	0.771–0.952	<0.001
SOFA score + CCL4	0.801	0.054	0.695–0.907	<0.001
APACHE II score + SOFA score + Pitt bacteremia score	0.854	0.045	0.766–0.943	<0.001
APACHE II score + SOFA score + Pitt bacteremia score + CCL4	0.885	0.039	0.809–0.960	<0.001
Procalcitonin	0.643	0.064	0.520–0.771	0.035
Procalcitonin + CCL4	0.645	0.069	0.510–0.779	0.036
Pitt bacteremia score + Procalcitonin	0.839	0.054	0.733–0.945	<0.001
SOFA score + Procalcitonin	0.783	0.055	0.675–0.892	<0.001
APACHE II score + Procalcitonin	0.713	0.063	0.588–0.837	0.002
APACHE II score + SOFA score + Pitt bacteremia score + Procalcitonin	0.856	0.044	0.769–0.942	<0.001

Receiver operating characteristic (ROC) curve analysis was performed to evaluate the predictive performance of clinical severity scores and CCL4 levels for ICU mortality. Abbreviations: AUC, area under the receiver operating characteristic curve; SE, standard error; CI, confidence interval; APACHE II, Acute Physiology and Chronic Health Evaluation II; SOFA, Sequential Organ Failure Assessment; CCL4, C-C motif chemokine ligand 4.

**Table 6:** Incremental AUC gains after addition of PCT and CCL4 to clinical severity scores.

Score/Model	Baseline AUC	AUC with PCT	ΔAUC (PCT)	AUC with CCL4	ΔAUC (CCL4)
APACHE II score	0.704	0.713	+0.009	0.737	+0.033
SOFA score	0.756	0.783	+0.027	0.801	+0.045
Pitt bacteremia score	0.827	0.839	+0.012	0.861	+0.034
APACHE II + SOFA + Pitt bacteremia score	0.854	0.856	+0.002	0.885	+0.031

ΔAUC represents the absolute increase in AUC obtained after the addition of either PCT or CCL4 to the corresponding baseline clinical severity model. Baseline AUC values were derived from models containing clinical severity scores alone. Positive ΔAUC values indicate improved discriminative performance for ICU mortality prediction. Abbreviations: AUC, area under the receiver operating characteristic curve; APACHE II, Acute Physiology and Chronic Health Evaluation II; SOFA, Sequential Organ Failure Assessment; PCT, procalcitonin; CCL4, C-C motif chemokine ligand 4.

**Table 7:** DeLong Test for Comparing ROC Curves of Prediction Models for ICU Mortality.

Comparison	AUC1	AUC2	ΔAUC	*p*-Value
Pitt bacteremia score vs. Pitt bacteremia score + CCL4	0.827	0.861	0.034	0.058
SOFA score vs. SOFA score + CCL4	0.756	0.801	0.046	0.012
APACHE II score vs. APACHE II score + CCL4	0.704	0.737	0.033	0.004
Clinical scores vs. Clinical scores + CCL4	0.854	0.885	0.031	0.041

The DeLong test was used to compare the differences between the areas under correlated ROC curves (ΔAUC) to evaluate whether the addition of CCL4 significantly improved the predictive performance of the clinical models for ICU mortality. Abbreviations: AUC, area under the receiver operating characteristic curve; ΔAUC, difference between AUC values; APACHE II, Acute Physiology and Chronic Health Evaluation II; SOFA, Sequential Organ Failure Assessment; CCL4, C-C motif chemokine ligand 4.

**Table 8:** Net Reclassification Improvement (NRI) and Integrated Discrimination Improvement (IDI) for Prediction of ICU Mortality.

Comparison	NRI	*p*-Value	IDI	*p*-Value
Pitt bacteremia score → Pitt bacteremia score + CCL4	0.182	0.038	0.041	0.027
SOFA score → SOFA score + CCL4	0.214	0.021	0.052	0.018
APACHE II score → APACHE II score + CCL4	0.197	0.029	0.047	0.022
Clinical scores → Full model (clinical scores + CCL4)	0.163	0.041	0.036	0.031

Net reclassification improvement (NRI) and integrated discrimination improvement (IDI) analyses were performed to evaluate whether the addition of CCL4 improved the predictive performance of the clinical scoring systems for ICU mortality. Abbreviations: NRI, net reclassification improvement; IDI, integrated discrimination improvement; APACHE II, Acute Physiology and Chronic Health Evaluation II; SOFA, Sequential Organ Failure Assessment; CCL4, C-C motif chemokine ligand 4.

## Data Availability

The datasets used and/or analyzed during the current study are available from the corresponding author on reasonable request.

## Supplementary Materials

The following supporting information can be downloaded at https://www.mdpi.com/article/10.3390/biomedicines14061390/s1: Figure S1: Receiver operating characteristic (ROC) curves comparing the predictive performance of clinical severity scores, CCL4, and combined prediction models for intensive care unit mortality; Table S1: Additional laboratory and physiological parameters according to ICU mortality; Table S2: Comparison of predictive performance of CCL4 and clinical scores for total mortality; Table S3: Correlation analysis between CCL4 and clinical and laboratory parameters; Table S4: Univariable and multivariable logistic regression analysis for predictors of MAKE30, Table S5. Distribution of Infection Sources, Culture Results, and Isolated Microorganisms Among Patients with Sepsis.

## Abbreviations

The following abbreviations are used in this manuscript:

AKI	Acute kidney injury
ALT	Alanine aminotransferase
APACHE II	Acute Physiology and Chronic Health Evaluation II
ARDS	Acute respiratory distress syndrome
AST	Aspartate aminotransferase
AUC	Area under the curve
BUN	Blood urea nitrogen
CCL2	C-C motif chemokine ligand 2
CCL3	C-C motif chemokine ligand 3
CCL4	C-C motif chemokine ligand 4
CCL14	C-C motif chemokine ligand 14
CI	Confidence interval
CKD	Chronic kidney disease
CLD	Chronic liver disease
CRP	C-reactive protein
EDTA	Ethylenediaminetetraacetic acid
ELISA	Enzyme-linked immunosorbent assay
FiO_2_	Fraction of inspired oxygen
GCS	Glasgow Coma Scale
GFR	Glomerular filtration rate
ICU	Intensive care unit
IDI	Integrated discrimination improvement
INR	International normalized ratio
IQR	Interquartile range
KDIGO	Kidney Disease: Improving Global Outcomes
LAG-3	Lymphocyte activation gene-3
LDH	Lactate dehydrogenase
MAKE30	Major adverse kidney events at 30 days
MIP-1α	Macrophage inflammatory protein-1 alpha
MIP-1β	Macrophage inflammatory protein-1 beta
NRI	Net reclassification improvement
NT-proBNP	N-terminal pro-B-type natriuretic peptide
NUTRIC	Nutrition Risk in the Critically Ill
OR	Odds ratio
PaO_2_	Partial pressure of oxygen
ROC	Receiver operating characteristic
SD	Standard deviation
SOFA	Sequential Organ Failure Assessment
Th	T helper
