# Supplementary material for: Association of Circulating C-C Motif Chemokine Ligand 4 to Disease Severity and Clinical Outcomes in Sepsis: A Prospective Observational Study
Source: Biomedicines. 2026 Jun 19;14(6):1390. doi: 10.3390/biomedicines14061390 (PMC13297423; doi:10.3390/biomedicines14061390)
Supplement: Supplementary file 1 [file biomedicines-14-01390-s001.zip › biomedicines-4308565-supplementary.pdf]

**Table S1.** Additional laboratory and physiological parameters according to ICU mortality

| Variable (Unit) | Total (n=75) | Survivors (n=46) | Non-survivors (n=29) | p-value |
|---|---|---|---|---|
| Uric acid (mg/dL) | 6.3 (4.1–9.3) | 6.2 (4.1–8.6) | 7.2 (4.9–10.5) | 0.194 |
| T3 (pg/mL) | 1.5 (1.2–2.0) | 1.4 (1.3–1.9) | 1.6 (1.0–2.25) | 0.927 |
| T4 (μg/dL) | 11.7 ± 3.7 | 12.4 ± 3.5 | 10.6 ± 4.0 | 0.065 |
| Ferritin (ng/mL) | 383.5 (202.8–726) | 301 (185–617.5) | 476 (264–1633) | 0.096 |
| Folate (ng/mL) | 5.65 (2.88–10.13) | 6.05 (3.25–10.1) | 5.6 (2.9–10.0) | 0.573 |
| Vitamin B12 (pg/mL) | 594 (369.5–807) | 490 (369.5–807) | 646 (373–803) | 0.482 |
| IL-6 (pg/mL) | 69.5 (32.2–323) | 48.9 (32.2–323) | 83.5 (54.2–129) | 0.661 |
| MPV (fL) | 10.7 ± 1.3 | 10.8 ± 1.4 | 10.5 ± 1.2 | 0.370 |
| RBC ($\times 10^6$/μL) | 3.89 ± 0.78 | 3.89 ± 0.89 | 3.90 ± 0.60 | 0.927 |
| Fibrinogen (mg/dL) | 4137 (3186–5146) | 3850.5 (3167–4773.5) | 4510 (3470.5–6240) | 0.136 |
| D-dimer (ng/mL FEU) | 4930 (1760–16400) | 4840 (1590–16000) | 5460 (3590–20400) | 0.340 |
| $PaO_2$ (mmHg) | 93.7 ± 1.6 | 93.8 ± 1.5 | 93.6 ± 1.8 | 0.512 |
| $FiO_2$ (%) | 29 (IQR 4) | 40 (IQR 7) | 40 (IQR 7) | 0.402 |

**Table S2.** Comparison of Predictive Performance of CCL4 and Clinical Scores for Total Mortality

| Model | AUC (95% CI) | ROC p-value | ΔAUC | Nagelkerke $R^2$ | Model p-value | Correct Classification (%) |
|---|---|---|---|---|---|---|
| CCL4 alone | 0.662 (0.529–0.795) | **0.017** | — | 0.130 | **0.006** | 66.2 |
| Pitt bacteremia score alone | 0.811 (0.707–0.916) | **<0.001** | — | 0.411 | **<0.001** | 78.1 |
| Pitt bacteremia score + CCL4 | 0.848 | **<0.001** | +0.037 | 0.482 | **<0.001** | 80.8 |

| Model | AUC (95% CI) | ROC p-value | ΔAUC | Nagelkerke $R^2$ | Model p-value | Correct Classification (%) |
|---|---|---|---|---|---|---|
| Full model (Pitt bacteremia score + CCL4 + APACHE II + SOFA) | 0.860 (0.772–0.949) | **<0.001** | +0.049* | 0.501 | **<0.001** | 82.2 |

The predictive performance of CCL4 and clinical severity scores for **total mortality** was evaluated using receiver operating characteristic (ROC) analysis and logistic regression models.
ΔAUC represents the increase in the area under the ROC curve compared with the reference clinical model.
Nagelkerke $R^2$ reflects the explanatory power of the logistic regression models, and correct classification indicates the overall predictive accuracy of each model.
**Abbreviations:** AUC, area under the receiver operating characteristic curve; CI, confidence interval; APACHE II, Acute Physiology and Chronic Health Evaluation II; SOFA, Sequential Organ Failure Assessment; CCL4, C-C motif chemokine ligand 4.

**Table S3**. Correlation Analysis Between CCL4 and Clinical and Laboratory Parameters

| Variable | r | p-value | Interpretation |
|---|---|---|---|
| Blood urea nitrogen (BUN) | 0.041 | 0.729 | Not significant |
| Glomerular filtration rate (GFR) | -0.016 | 0.889 | Not significant |
| Procalcitonin | 0.025 | 0.829 | Not significant |
| Uric acid | 0.033 | 0.781 | Not significant |
| C-reactive protein (CRP) | 0.165 | 0.157 | Not significant |
| NT-proBNP | -0.103 | 0.568 | Not significant |
| Vasopressor requirement | 0.060 | 0.612 | Not significant |
| APACHE II score | 0.213 | 0.068 | Borderline, not significant |
| MAKE30 | 0.108 | 0.363 | Not significant |
| Pitt bacteremia score | 0.153 | 0.195 | Not significant |
| NUTRIC score | -0.046 | 0.697 | Not significant |

Correlation analysis was performed to evaluate the relationship between **CCL4 levels** and selected clinical and laboratory variables. Correlation coefficients (r) were calculated using **Pearson or Spearman correlation analysis**, as appropriate.
**Abbreviations:** BUN, blood urea nitrogen; GFR, glomerular filtration rate; CRP, C-reactive protein; NT-proBNP, N-terminal pro–B-type natriuretic peptide; APACHE II, Acute Physiology and Chronic Health Evaluation II; MAKE30, major adverse kidney events within 30 days; NUTRIC, Nutrition Risk in the Critically Ill score; CCL4, C-C motif chemokine ligand 4.

**Table S4.** Univariable and Multivariable Logistic Regression Analysis for Predictors of MAKE30

| Variables | Univariable OR | p | 95% CI | Multivariable OR | p | 95% CI |
|---|---|---|---|---|---|---|
| **BUN** | 1.059 | <0.001 | 1.028–1.090 | 1.047 | 0.008 | 1.012–1.083 |
| **Creatinine** | 2.760 | <0.001 | 1.616–4.715 | 1.952 | 0.037 | 1.040–3.663 |
| **CCL4** | 1.000 | 0.359 | 1.000–1.001 | — | — | — |
| **Albumin** | 0.416 | 0.053 | 0.171–1.011 | 0.322 | 0.046 | 0.106–0.978 |

OR: odds ratio; CI: confidence interval; BUN: blood urea nitrogen.
Variables with $p < 0.10$ in univariable analysis were included in the multivariable logistic regression model. Accordingly, BUN, creatinine, and albumin were entered into the multivariable model. CCL4 was not included in the multivariable analysis because it was not significant in univariable analysis. MAKE30 was defined as the composite outcome of mortality, initiation of renal replacement therapy, or persistent renal dysfunction within 30 days.

**Table S5.** Distribution of Infection Sources, Culture Results, and Isolated Microorganisms Among Patients with Sepsis

| **Variable** | **n (%)** |
|---|---|
| **Infection source** | |
| Urinary tract infection | 24 |
| Respiratory infection | 14 |
| Hepatobiliary infection | 8 |
| Soft tissue infection | 7 |
| Gastrointestinal infection | 3 |
| Unspecified / no clear focus recorded | Remaining patients |
| **Microbiological culture status** | |
| Culture-negative sepsis | 35 |
| Blood culture positive | 12 |
| Urine culture positive | 17 |
| Endotracheal aspirate (ETA) culture positive | 2 |
| **Most commonly isolated pathogens** | |

| | |
|---|---|
| *Escherichia coli* | 10 |
| *Klebsiella pneumoniae* | 11 |
| *Pseudomonas aeruginosa* | 2 |
| *Staphylococcus aureus* | 2 |
| *Staphylococcus epidermidis* | 2 |
| *Enterococcus faecium* | 1 |
| *Acinetobacter* spp. | 1 |
| *Candida albicans* | 2 |
| Non-*albicans Candida* species | 3 |

Abbreviations: ETA, endotracheal aspirate; ICU, intensive care unit.

**Figure S1.** Receiver operating characteristic (ROC) curves comparing the predictive performance of clinical severity scores, CCL4, and combined prediction models for intensive

care unit mortality.

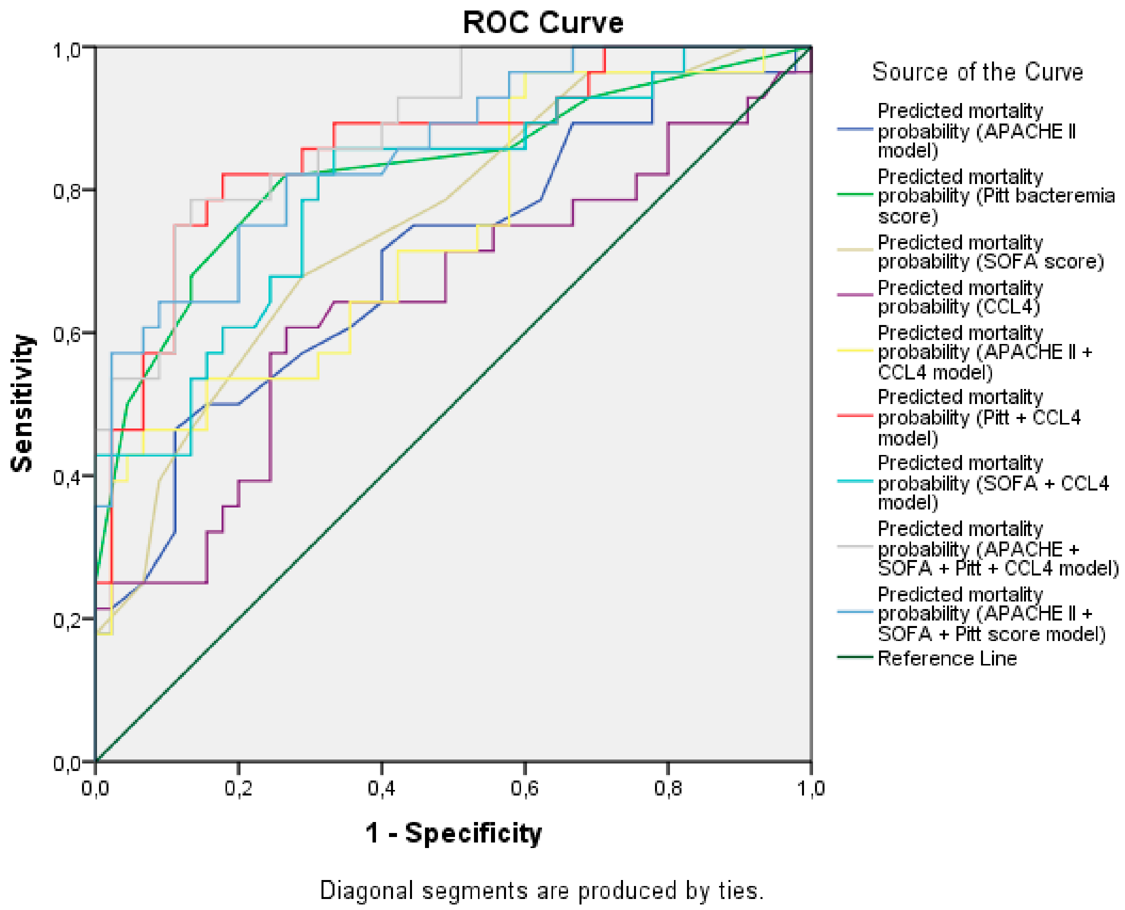


Receiver operating characteristic (ROC) curves were generated to evaluate the predictive performance of clinical severity scores, CCL4 levels, and combined prediction models for **intensive care unit (ICU) mortality**. The models included APACHE II score, Pitt bacteremia score, SOFA score, CCL4 alone, and their combined models. The diagonal line represents the line of no discrimination. Higher area under the curve (AUC) values indicate better discriminative performance of the prediction models.
**Abbreviations:** ROC, receiver operating characteristic; AUC, area under the curve; APACHE II, Acute Physiology and Chronic Health Evaluation II; SOFA, Sequential Organ Failure Assessment; CCL4, C-C motif chemokine ligand 4.
